# Potentially Synbiotic Yellow Mombin Beverages: Stability during Refrigerated Storage, Physicochemical Characteristics, and Sensory Properties

**DOI:** 10.3390/foods12101994

**Published:** 2023-05-15

**Authors:** Luis Eduardo Guieu Galvao Telles Ribeiro, Leonam da Silva Pereira Batista, Cristiane Fernandes de Assis, Karla Suzanne Florentino Silva Chaves Damasceno, Francisco Canindé de Sousa Júnior

**Affiliations:** 1 Programa de Pós-Graduação em Nutrição, Universidade Federal do Rio Grande do Norte, Av. Senador Salgado Filho, 3000, Natal 59078-970, RN, Brazil; luis.ribeiro.098@ufrn.edu.br (L.E.G.G.T.R.);; 2 Departamento de Nutrição, Universidade Federal do Rio Grande do Norte, Av. Senador Salgado Filho, 3000, Natal 59078-970, RN, Brazil; 3 Departamento de Farmácia, Universidade Federal do Rio Grande do Norte, R. Gal. Gustavo Cordeiro de Faria, s/n, Petrópolis, Natal 59012-570, RN, Brazil

**Keywords:** probiotic, prebiotic, fermentation, functional food, tropical fruit

## Abstract

This study aimed to develop potentially synbiotic yellow mombin (*Spondias mombin* L.) beverages added with fructooligosaccharides and *Lactiplantibacillus plantarum* NRRL B-4496. Six formulations of yellow mombin beverages were prepared to measure the influence of fermentation and pH, which was adjustment to 4.5 for stability and quality parameters. Formulations were evaluated for probiotic survival, pH, titratable acidity, total phenolic compounds (TPC), and antioxidant activity for 28 days at 4 °C. Additionally, the proximate composition, color, sensory aspects, and survival to simulated gastrointestinal conditions were studied. At 21 days of storage, the viability of *L. plantarum* was 9 CFU/mL for the fermented symbiotic (SYNf) and non-fermented symbiotic with adjusted pH (SYNa) formulations. In addition, the fermented synbiotic with an adjusted pH beverage (SYNfA) showed a count of 8.2 log CFU/mL at 28 days. The formulations showed a high TPC (234–431 mg GAE/L), antioxidant activity (48–75 µM trolox), and a potential use as low-calorie beverages. The SYNf formulation showed an acceptability index higher than 70% and a high purchase intent. The SYNf and SYNa formulations maintained suitable probiotic counts after exposure to the simulated gastrointestinal digestion. Therefore, it was possible to develop a new potentially synbiotic yellow mombin beverage with a high sensory acceptance, supplying the market with a new functional food alternative.

## 1. Introduction

Probiotics are living microorganisms that provide beneficial effects to consumers when administered in adequate amounts [1]. Probiotics can generate a great variety of positive health effects, such as the regulation of gastrointestinal microbiota, immune system modulation, improvement in lipid profile, insulin sensibility, and a protective effect against symptoms of inflammatory bowel disease, irritable bowel syndrome, atopic dermatitis, type 1 diabetes, COVID-19, and others [2,3,4,5,6].

Studies observed a rising growth in the search for probiotic products with yogurts and fermented milks as the main probiotic products available in the market nowadays [7]. However, it is estimated that around 65% of the world population will develop lactose intolerance at some point in life, and in addition to that, an increase in the search for a plant-based diet is a growing reality for many individuals, leading to an estimated market value of 14.5 billion dollars by 2050 [8].

Fruit juices added with probiotics showed promising results [9]. Fruit juices are rich in nutrients, vitamins, and antioxidant compounds, and have high amounts of fermentable sugars, providing suitable conditions for probiotic growth [10,11], in addition to having a refreshing flavor and being able to be consumed by individuals of all ages [10]. Different probiotic strains have been successfully inoculated in a wide variety of fruit juices, such as orange [12,13], pineapple and juçara (*Euterpe edulis* Martius) [14], jujube (*Zizyphus jujuba* Mill.) [2], and apple [11,15].

However, the maintenance in the viability of probiotics in fruit juices is difficult since these beverages have a low pH [16]. The viability can be also influenced by the storage temperature, the nutritional composition of food, and the probiotic strain used [17]. Furthermore, probiotic cultures can alter sensory characteristics of the product and can impair the acceptance of the product [18], which is essential to obtain a pleasing sensory profile for market success [10].

Fructooligosaccharides (FOS) are prebiotic fibers that can be fermented by intestinal bacteria, conferring positive effects to the host and being considered as a product with functional allegations [19]. FOS do not induce big changes in taste and texture profiles, enabling the formulation of products with high amounts of dietary and no significant changes on sensory acceptance in comparison to non-prebiotic beverages [16]. The simultaneous use of probiotics and prebiotics leads to the development of a synbiotic product, which may be able to improve probiotic stability, viability, and intestinal colonization [7,10,20]. Therefore, prebiotics (such as FOS) could be used to improve the stability of probiotic cultures in fruit juices.

Yellow mombin (*Spondias mombin* L.) is an elliptical-shaped fruit, 3–4 cm in length, and belongs to the Anacardiaceae family [21]. This fruit exhibits high tannin, vitamin C, and carotenoid contents [21,22]. This composition has shown both gastroprotective and ulcer healing capabilities [23], protective effects against ovarian cancer cells [24], and reduced ventricular remodeling post-myocardial infarction [25]. Yellow mombin fruit is mostly found in domestic orchards in the North and Northeast regions of Brazil and is considered a part of the socio-biodiversity of these regions [26,27]. Yellow mombin juice can be a suitable matrix for the development of novel probiotic and synbiotic foods. However, it is essential to evaluate the influence of adding any substance in a food matrix, since any change can compromise the physicochemical, microbiological, and sensory aspects of new foods [17].

Therefore, this study aimed to develop a potentially synbiotic yellow-mombin beverages, using FOS and *Lactiplantibacillus plantarum* NRRL B-4496. The stability of the fermented and non-fermented formulations was evaluated during the refrigerated storage. In addition, proximate composition, color, sensorial evaluation, and probiotic survival under simulated gastrointestinal conditions were studied.

## 2. Materials and Methods

### 2.1. Materials and Chemicals

The FOS were purchased from NewNutrition (Ribeirão Preto, SP, Brazil). The frozen yellow mombin pulp was obtained from Ster Bom (Macaíba, RN, Brazil). The De Man, Rogosa and Sharpe (MRS) broth was purchased from Becton, Dickinson and Co. (Franklin Lakes, NJ, USA). The gallic acid, Folin–Ciocalteu phenol reagent, DPPH (2,2-diphenyl-1-picrilhidrazil), trolox (6-Hydroxy-2,5,7,8-tetramethylchroman-2-carboxylic acid), pepsin, bile salts, and pancreatin were purchased from Sigma-Aldrich (Saint Louis, MO, USA). All other reagents used were of analytical grade.

### 2.2. Probiotic Strain

*Lactiplantibacillus plantarum* NRRL B-4496 was provided by the ARS Culture Collection—NRRL (U.S. Department of Agriculture, Peoria, IL, USA). The strain was stored at −20 °C in microtubes containing the MRS broth and 15% glycerol (*w*/*v*).

### 2.3. Inoculum Preparation

For the inoculum preparation, 1 mL of the glycerol stock culture was transferred to 50 mL of the MRS broth and incubated (model Q816M20, Quimis, Diadema, SP, Brazil) at 37 °C for 24 h. Then, 5 mL of the initial culture was transferred to 50 mL of the MRS broth and incubated at 37 °C for 18 h [28]. The cells were centrifuged at 2000 rpm (model 206 BL II, Fanem, Guarulhos, SP, Brazil) for 10 min, washed with NaCl 0.9% (*w*/*v*), and subjected to a second centrifugation. The inoculum was obtained by the addition of 5 mL of NaCl 0.9% to the pellet and was used for both the fermented and unfermented beverages [17].

### 2.4. Obtaining Potentially Synbiotic Yellow Mombin Beverages

The six formulations were prepared: CON (control), PREB (prebiotic beverage), SYN (unfermented synbiotic beverage), SYNf (fermented synbiotic beverage), SYNa (unfermented synbiotic beverage with a pH adjusted to 4.5), and SYNfa (fermented synbiotic beverage with a pH adjusted to 4.5) as shown in Table 1.

The yellow mombin formulations were prepared using 100 g of frozen pulp for 200 mL of beverage. The pH of the SYNa and SYNfa formulations was adjusted to a pH 4.5 using NaOH 1.0 M. The formulations were pasteurized at 80 °C for 15 min with a subsequent thermal shock using an ice bath for 5 min. The inoculum (10% *v*/*v*) (Section 2.3) was aseptically added to the pasteurized juice to obtain the non-fermented beverages (SYN and SYNa). After adding the inoculum (10% *v*/*v*) for the fermented beverages (SYNf and SYNfa), the formulations were incubated at 30 °C for 18 h [18]. The fructooligosaccharides powder was added for a final concentration of 25 g/L.

### 2.5. Stability of Potentially Synbiotic Beverages during Refrigerated Storage

All the beverage formulations obtained were dispersed in sterile tubes at 4 °C. The viable count, pH, titratable acidity, total soluble solids (TSS), total phenolic compounds (TPC), and antioxidant activity were recorded before storage and at intervals of 7 days for 28 days. Next, a *L. plantarum* count was performed in the MRS agar for serial dilutions up to 10^−10^ and incubated at 37 °C for 72 h [18]. The pH was determined using a digital potentiometer (model Luca-210, Lucadema, São José do Rio Preto, SP, Brazil). The titratable acidity was determined by titration with phenolphthalein and 0.01 mol/L NaOH. The level of TSS (°Brix) was obtained using a refractometer (model Q767BD, Quimis). The TPC was determined by the Folin–Ciocalteu method adapted to 96 well microplates [29]. The absorbance was read at 750 nm (model EPOCHH, BioTek, Campinas, SP, Brazil), and the results were expressed as milligrams of gallic acid equivalent per liter (mg GAE/L). The antioxidant activity was evaluated by the DPPH radical scavenging, according to Nóbrega et al. [30].

### 2.6. Proximate Composition

The proximate composition was evaluated on the potentially synbiotic beverages with a probiotic survival for at least 21 storage days and controls (CON and PREB). The measurements of the moisture, ash, lipids (adapted to liquid samples with the addition of 5 g of sample weighed in a cotton ball inside a Soxhlet cartridge), and protein (using 6.25 as a conversion factor) were carried out according to the AOAC methods [31]. The fiber content was estimated based on the nutritional information of the ingredients (Frozen yellow mombin pulp and FOS), and the carbohydrates were obtained by the difference. The caloric value (Kcal) was calculated using the Atwater conversion factors (protein × 4, carbohydrate × 4, and lipid × 9).

### 2.7. Color Determination

The L*, a*, and b* coordinates were obtained using a digital colorimeter (model WR10, Shenzhen Wave Optoelectronics Technology Co. Ltd., Shenzhen, Guangdong, China). The color difference (ΔE) between CON and the other formulations was calculated according to Equation (1):

(1) $$\Delta E=\sqrt{{\left(\Delta L^{*}\right)}^{2}+{\left(\Delta a^{*}\right)}^{2}+{\left(\Delta b^{*}\right)}^{2}}$$

### 2.8. Sensory Evaluation

The sensory evaluation was carried out using 91 untrained panelists, including students and staff of UFRN (74% female, 71% with an age range of 20–26 years). The beverages were coded with random three-digit numbers and presented in a sequential monadic way in individual plastic cups. Water and salty biscuits were provided for palate cleansing. The overall acceptance of the beverages was evaluated using the nine-point hedonic scale, ranging from “extremely disliked” (score 1) to “extremely liked” (score 9) [32]. The acceptability index (AI) was calculated using Equation (2):

(2) $$AI\left(\%\right)=\left(\frac{Average\;grade\;given\;to\;the\;formulation}{9}\right)\times 100$$

The sensory attributes of acidity, sweetness, viscosity, and color were evaluated using a just about right (JAR) scale, ranging from “less intense than I like” (score 1) to “more intense than I like” (score 5), with a score of 3 being “ideal, the way I like”. In addition, the consumer attitude concerning the purchase intent was evaluated with a five-point scale ranging from “I would certainly not buy” (score 1) to “I would certainly buy” (score 5) [33]. This study was approved by the Ethics Committee of the UFRN (Certificate of Presentation for Ethical Assessment—CAAE no. 07811118.9.0000.5292). All volunteers signed an informed consent form to participate in the study.

### 2.9. Simulated Gastrointestinal Conditions

The survival of *L. plantarum* under simulated gastrointestinal conditions was evaluated in vitro according to Matias et al. [34] with adaptations. The SYNf and SYNa formulations were submitted to the gastric and intestinal simulated conditions. Sodium chloride 0.9% inoculated with a probiotic culture was used as a control. The gastric phase fluid consisted of pepsin (2500 U/mL) was diluted in 9 mL of NaCl 0.9% with the pH adjusted to 3.0. The intestinal fluid consisted of pancreatin (800 U/mL) and bile salts (625 μL at 4% *w*/*v*) was diluted in 9 mL of NaCL 0.9% with the pH adjusted to 7.0. For the gastric phase (GP) 1 mL of each formulation was added to the gastric fluid and incubated at 37 °C and 200 rpm for 2 h. For the intestinal phase (IP), 1 mL of each GP fluid was added to the intestinal fluid and incubated in the same conditions. The enumeration of the viable counts was performed before the simulation and after each simulated gastrointestinal phase.

### 2.10. Statistical Analysis

The experiments and measurements were performed in triplicate and the results were expressed as arithmetic mean ± standard deviation. The statistical analysis of the beverage characterizations and the simulated gastrointestinal conditions was performed using the one-way ANOVA with Tukey’s post-test using the Statistica 7.0 software program (StatSoft Inc., Tulsa, OK, USA). The sensory analysis results were expressed as median, and the non-parametric Kruskal–Wallis test with the Dunn test was performed for multiple comparisons along with Bonferroni. The data obtained by the JAR test were compared to the overall acceptance data with a penalty test to determine which attributes significantly impacted acceptance. The sensory analysis data statistics were carried out using the XLSTAT statistical package (Addionsoft, Paris, France). All the statistical analyses considered a significance level of 95% (*p* < 0.05).

## 3. Results and Discussion

### 3.1. Stability of Potentially Synbiotic Beverages during Refrigerated Storage

The viability of *L. plantarum* NRRL B-4496 in potentially synbiotic yellow mombin beverages showed a count above 8 log CFU/mL for SYNf and SYNa at 21 days and 28 days, respectively, (Table 2). The literature has shown that a reduction in probiotic viability in refrigerated fruit juices occurs due to a low pH [16]. The cell count decay in the SYNf and SYNa formulations after 21 days could have been caused by a drop in the *L. plantarum* metabolic activity, as well as the production of metabolites and bacteriocins, impairing the viability of the probiotics [35].

The results indicated that the presence of the FOS as an additional carbon source and the pH adjustments in both the SYNa and SYNfa were possibly positive determinants for the probiotic survival in the yellow mombin beverages during the refrigerated storage, as we could observe the beneficial influence of both adaptations individually and simultaneously [36,37]. A study with cornelian cherry juice (*Cornus mas* L.) showed an increase in viability for *Lacticaseibacillus casei* T4 after pH adjustments for 28 days [38].

After 28 days of storage, the SYNfa formulation showed probiotic counts higher than those previously described [39,40]. For example, *Lactobacillus acidophilus* La5 in fermented yacon beverages with 5.97 log CFU/mL at 4 °C for 27 days of storage [39] and *Limosilactobacillus reuteri* ATCC 55730 in fermented almond beverages with 7.06 log CFU/mL after 28 days of storage [40]. In comparison to studies using vegetable beverages, both SYNf and SYNa showed similar or higher viable counts in comparison to the 21 day storage time [36,39,40]. Therefore, the fermented and/or pH-adjusted yellow mombin juice added with FOS could be considered a suitable vehicle for inoculation and maintenance of *L. plantarum* viability.

The SYN formulation presented low probiotic stability, losing its viability on storage day 7. The probiotic possibly did not adapt to the unfermented food matrix, as well as the very acidic pH. The adaptability of the microorganism to the medium is of fundamental importance for its development [37].

The physicochemical and antioxidant stability results are described in Table 3. The SYNf formulation was able to keep a stable pH during the whole storage period while the remaining formulations showed a slight increase from day 7 to day 21, followed by a decrease at day 28. Khezri et al. [37] observed a decrease in the pH in fig juice formulations added with inulin and *Lactobacillus delbrueckii* DSMZ 15996, while no significant difference was observed in orange juice with added FOS and *L. paracasei* throughout its storage. The differences found in this study could be caused by the different organic acid profiles, soluble solids content, nutritional availability, and the original matrix pH [37].

The titratable acidity for all the formulations showed a slight decrease at day 28, which could be related to the natural degradation of the citric acid or the decrease in the production of lactic acid related to the loss of viability in SYNf and SYNa [37]. However, the decrease in titratable acidity was not reflected in the pH of the formulations on day 28. This could have occurred because of the right buffering of the organic acids in the beverage, avoiding pH altering even with acid profile changes [41,42].

The TSS results demonstrated similar variations within all the formulations with an increase at day 7, possibly due to the acid hydrolysis of the complex sugars, which could relate to the increase in TSS and pH at the same time [36,43,44]. A similar pattern could be observed in an orange juice and hibiscus tea mixed beverage with added *L. casei* 01 and FOS [44]. A continuous acid hydrolysis can lead to the breaking down of sugars into acids, reducing both the TSS and pH values [44]. As the presence of FOS lead to an increase in the TSS, the results for the CON were significantly lower (*p* < 0.05).

The TPC showed different variations for each formulation, but the results from days 0 and 28 were statistically equal in all the formulations except SYN. The formulations fermented by LAB are expected to present higher TPC values, since those bacteria can produce enzymes, such as β-glucosidase, amylase, and cellulase, leading to the hydrolysis of vegetable wall cells and favoring the release of free phenolics [45,46]. It has been observed that an extract produced from the peel and seed of yellow mombin has around 1149 mg GAE/L [47]. Since the peel and seed are rich in phenolic compounds and represent about 50% of the total fruit content, the industrial frozen pulp may suffer from enzymatic and/or chemical oxidation due to the exposure to industrial processing and lingering storage periods; therefore, some decrease in TPC can be expected in comparison to the values obtained from fresh fruit (260.2 mg GAE/100 g) [18,48,49,50]. Nonetheless, all the values obtained in this study are at least twofold the values obtained for the yellow mombin fermented beverage with a pH adjusted to 6.4 (94.9 mg GAE/L) [18], similar to apple (339 mg GAE/L) and pineapple (358 mg GAE/L) juices [49]. Furthermore, phenolic compounds are secondary metabolites produced by plants, which can be found in most fruits and have high antioxidant and disease-prevention potential [51]. Since animal products do not exhibit polyphenol content, the obtained formulations showed a commercial advantage over dairy beverages [41,46,51,52].

The values obtained for the antioxidant activity for PREB, SYNf, SYNa, and SYNfa at days 0 and 28 can be related to TPC values, as phenolics acids are mainly responsible for the DPPH scavenging activity [53]. A higher reduction in antioxidant activity could be observed in the control sample in comparison to the synbiotic beverage of yacon, which can be due to the production of antioxidant compounds, such as polyphenols, flavonoids, and β-carotenes through lactic fermentation [39]. Different probiotic strains may have different behaviors and strains from the same species may have different metabolisms in the same matrix [41,54]. Thus, the obtained results from this study may be specific to the strain and matrix used. In turn, the results may indicate possible protection and even increased production of antioxidant compounds by the action of lactic fermentation metabolites, as well as mechanical protection provided by the prebiotics [39,45].

### 3.2. Proximate Composition

The formulations of potentially synbiotic beverages with probiotic survival for at least 21 storage days and controls (CON and PREB) were evaluated for proximate composition. The results can be observed in Table 4. 

The moisture value for CON was higher than the other formulations. As expected, the addition of the FOS led to an increase in TSS and a decrease in moisture [49]. Lipids were not detected only in control formulations. This can be due to the presence of cellular components of probiotics, as well as a possible production of fatty acids through lactic fermentation [55,56]. The energetic value of a 200 mL portion ranged from 33 to 56 Kcal (PREB and SYNfa, respectively), representing from 1.65% to 2.8% of the daily consumption of 2000 Kcal [57]. Thus, these formulations can be considered low-calorie beverages and of interest to individuals with caloric restrictions [16]. The results were similar to a cajuí (*Anacardium othonianum* Rizz.) beverage added with *Saccharomyces boulardii*-17, with about 32 Kcal in 200 mL of unsweetened beverage [58].

The estimated value for fiber in the formulations with the addition of FOS (3 g/100 g) represented about 12% of the recommended 25 g of the daily consumption of 2000 Kcal [59], as well as being at least twofold higher than the values obtained for an almond milk synbiotic beverage (1.2 g/100 g) [40].

The values obtained for sugars ranged from 1.8 to 2.7 g/100 g for the SYNfa and SYNf formulations, respectively, being similar to values found in unsweetened cajuí beverages (2.9 g/100 g) [58], and lower values in comparison to an orange juice and hibiscus tea mixed beverage (4.6 g/100 g) [44]. The low sugar content added to the antioxidant effect of the phenolic compounds, the presence of the FOS as a bioactive fiber, and a possible hypoglycemic effect by the probiotics make the obtained formulations viable for consumption for diabetic individuals [15,37,52,58].

### 3.3. Color

All color coordinates (L*, a*, and b*) of the formulations with an adjusted pH (SYNa and SYNfa) showed significant differences in comparison to the unadjusted ones (Table 5). The color difference observed for the adjusted pH formulations may have been due to the addition of NaOH as observed by Ribeiro et al. [18], since it led to lower L* values and higher a* and b* values. 

The results for the ΔE showed that the SYNf showed the closest color coordinates to CON among the other formulations. Moreover, the SYNa and SYNfa values did not show significant difference between them but both showed higher values than the SYNf. 

Color is a fundamental parameter for the acceptance of beverages. Thus, PREB, SYNf, and SYNa were selected for the sensory analysis. The PREB was used as a standard formulation, hence its similar characteristics to CON and the functional difference of the addition of the FOS. The SYNf exhibited a low ΔE value, being a good synbiotic alternative for the sensory tests. Both the SYNf and SYNa kept a high viable cell count for 21 days and each presented a different method used for the probiotic adaptation to the beverage, being of interest to compare the acceptance of each method.

### 3.4. Sensory Evaluation

The averages of the overall acceptance scores provided by the panelists were 7.03 ± 1.73, 6.79 ± 1.83, and 4.74 ± 2.14 for PREB, SYNf, and SYNa formulations, respectively, (Table 6). The PREB and SYNf formulations were well accepted [59] with an AI of 78.14% and 75.46%, respectively, with a statistical difference between both medians (*p* < 0.05). On the other hand, the AI of SYNa was considered low (<70.0%) [59]. 

The results obtained from the JAR test showed that over 50% of the panelists considered all the sensory attributes evaluated as ideal for the PREB and SYNf formulations (Tables S1–S3—Supplementary Material). Meanwhile, the only attribute for the SYNa formulation, with over 50% of the panelists considering it ideal, was the color. The results, according to the overall acceptance results, show the SYNa formulation had the lowest results, with a high percentage of the panelists considering it “too weak” or “too strong” as one the attributes.

The Supplementary Material (Tables S1–S3) presents the penalties obtained for each formulation. All the attributes were considered significant for the overall acceptance of the three formulations. The attributes for the PREB and SYNf formulations which negatively impacted the acceptance were too strong in acidity (29.67% and 30.77%, respectively) and too weak in sweetness (36.26% and 39.56%, respectively). The viscosity and color of both the formulations were considered ideal by most of the panelists. 

On the other hand, SYNa was severely penalized by all the sensory attributes: too weak acidity (75.82%), too weak in sweetness (27.47%), too strong in sweetness (47.25%), too strong in viscosity (41.76%), and a too strong darker color (41.76%). All these attribute penalties could be related to the pH adjustment. Adjusting the pH of the yellow mombin juice to 4.5 may have caused changes in color, viscosity, and flavor by adding NaOH [19]. The low acidity was perceived by panelists who related it to be too strong in sweetness. 

**Figure 1:**
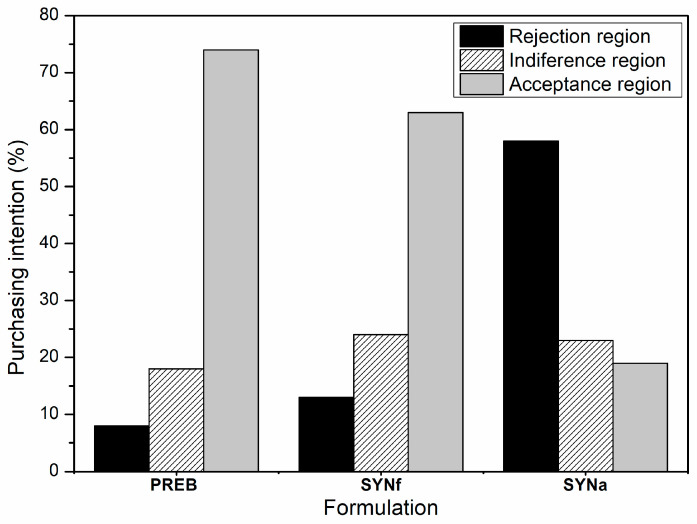
Purchase intention for prebiotic (PREB), fermented symbiotic (SYNf), and unfermented symbiotic with adjusted pH (SYNa) yellow mombin beverages.

The PREB and SYNf formulations exhibited high mean values of purchase intent (3.99 ± 1.04 and 3.77 ± 1.16, respectively), with 74% and 63% of the panelists reporting in the acceptance region (Figure 1). On the other hand, the SYNa formulation exhibited a mean purchase intent of 2.43 ± 1.12, with 58% of the panelists reporting in the rejection region.

The sensory evaluation results indicated that the potentially symbiotic formulation SYNf presented higher acceptance values than other non-dairy probiotic or symbiotic beverages, such as Brazil nut [36], okara [7], sugarcane [49], guava (*Psidium guajava* L.) [17], and yellow mombin with the pH adjusted to 6.4 [18]. Therefore, SYNf exhibited a good overall acceptance and could be viable for commercialization.

### 3.5. Simulated Gastrointestinal Conditions

Table 7 presents the viability of *L. plantarum* in potentially symbiotic beverages before and after each simulated gastrointestinal phase. Both the SYNf and SYNa formulations showed no significant differences along the simulation process, maintaining counts above 8 log CFU/mL, while the control formulation (NaCl 0.9% with probiotic) decreased its count, showing a possible protective effect from the matrix. Similar results were obtained by Ribeiro et al. [18] who did not observe significant changes in *L. acidophilus* NRRL B-4495 viability after exposure to simulated gastrointestinal conditions. The presence of the FOS may also have a mechanical protective effect on the bacteria similar to cricket protein hydrolysates as found by Dridi et al. [35] in a commercial beverage with *L. acidophilus* CL1285, *L. casei* LBC80R and *Lacticaseibacillus rhamnosus* CLR2, maintaining a survival rate above 97% for both gastric and intestinal phases.

The probiotic survival observed was higher than that found for *L. rhamnosus* ATCC 7469 in guava juice [17] and *L. reuteri* ATCC 55730 in a fermented almond beverage [40], with survival rates of 55% and 51%, respectively. The specific conditions of fermentation and food matrix employed in beverage formulation can also affect the maintenance of the probiotic stability [34]. Another possible factor is the naturally low pH of yellow mombin juice, providing conditions for adaptive stress, enhancing the strain tolerance to acidic environments, and to gastrointestinal conditions by stimulating the development of resistance mechanisms, such as the maintenance of cell membrane functionality and intracellular pH [17,35,36].

## 4. Conclusions

Yellow mombin juice is a suitable matrix for incorporating *L. plantarum* NRRL B-4496 as a probiotic and FOS as a prebiotic, resulting in potentially synbiotic formulations with high probiotic survival during refrigerated storage. The formulations also showed stable antioxidant activity and potential use as a low-calorie beverage, which confers nutritional benefits to this functional food. The SYNf formulation showed a higher acceptability index of 70% in the sensory evaluation, meaning that SYNf had a better acceptance among the synbiotic formulations. The SYNf formulation also showed high purchase intent, suggesting good perspectives for possible commercialization, as well as high survival in the simulated gastrointestinal conditions alongside SYNa. Therefore, it was possible to develop a potentially synbiotic yellow mombin beverage, supplying the market with a new functional food alternative using a Brazilian socio-biodiversity fruit.

## Figures and Tables

**Table 1:** Composition and probiotic adaptation strategies used in yellow-mombin beverage formulations.

Formulation	FOS (25 g/L)	*L. plantarum* (10% *v*/*v*)	Fermentation (30 °C, 18 h)	pH Adjustment (4.5)
CON	-	-	-	-
PREB	X	-	-	-
SYN	X	X	-	-
SYNf	X	X	X	-
SYNa	X	X	-	X
SYNfa	X	X	X	X

Notes: CON—control juice; PREB—prebiotic beverage; SYN—unfermented symbiotic; SYNf—fermented symbiotic; SYNa—unfermented synbiotic with adjusted pH; SYNfA—fermented synbiotic with adjusted pH.

**Table 2:** *L. plantarum* viability in potentially symbiotic beverages stored at 4 °C for 28 days.

Parameter	Storage Time (Days)	Formulation			
		SYN	SYNf	SYNa	SYNfA
Viability (log CFU/mL)	0	9.0 ± 0.4 ^Aa^	9.2 ± 0.2 ^Aa^	9.1 ± 0.9 ^Aa^	9.6 ± 1.6 ^Aa^
	7	<2 ^Bb^	9.8 ± 1.3 ^Aa^	8.7 ± 0.0 ^Aa^	9.7 ± 0.1 ^Aa^
	14	<2 ^Bb^	8.2 ± 0.9 ^Aa^	9.5 ± 0.3 ^Aa^	10.1 ± 1.7 ^Aa^
	21	<2 ^Bb^	9.0 ± 1.0 ^Aa^	9.0 ± 2.3 ^Aa^	11.5 ± 0.1 ^Aa^
	28	<2 ^Bb^	<2 ^Bb^	<2 ^Bb^	8.2 ± 0.1 ^Aa^

Notes: SYN—unfermented symbiotic; SYNf—fermented symbiotic; SYNa—unfermented synbiotic with adjusted pH; SYNfA—fermented synbiotic with adjusted pH. ^A,B^ Values with the same uppercase letter in a row denotes that the formulations do not differ significantly at the same storage time (*p* < 0.05). ^a,b^ Values with the same lowercase letter in a column denotes that the same formulation does not differ significantly over storage time (*p* < 0.05).

**Table 3:** Physicochemical and antioxidant stability of yellow mombin beverages during 28 days of storage.

Parameter	Storage Time (Days)	Formulation					
		CON	PREB	SYN	SYNf	SYNa	SYNfA
pH	0	2.4 ± 0.0 ^Bb^	2.4 ± 0.0 ^Bb^	2.4 ± 0.1 ^Bb^	2.3 ± 0.1 ^Ba^	4.5 ± 0.0 ^Ab^	4.4 ± 0.1 ^Abc^
	7	2.5 ± 0.0 ^Bab^	2.4 ± 0.0 ^Bab^	2.5 ± 0.0 ^Ba^	2.4 ± 0.1 ^Ba^	4.6 ± 0.0 ^Aa^	4.7 ± 0.1 ^Aa^
	14	2.5 ± 0.1 ^Ba^	2.5 ± 0.1 ^Ba^	2.5 ± 0.0 ^Ba^	2.5 ± 0.1 ^Ba^	4.7 ± 0.1 ^Aa^	4.8 ± 0.1 ^Aa^
	21	2.4 ± 0.0 ^Cb^	2.4 ± 0.0 ^BCab^	2.5 ± 0.0 ^Ba^	2.5 ± 0.1 ^BCa^	4.6 ± 0.1 ^Aa^	4.6 ± 0.0 ^Aab^
	28	2.2 ± 0.1 ^Cc^	2.2 ± 0.1 ^BCc^	2.3 ± 0.1 ^Bb^	2.4 ± 0.0 ^Ba^	4.3 ± 0.0 ^Ac^	4.3 ± 0.0 ^Ac^
Titratable acidity (mg citric acid/mL)	0	0.79 ± 0.01 ^Aab^	0.77 ± 0.01 ^Aab^	0.78 ± 0.02 ^Aab^	0.79 ± 0.01 ^Aab^	0.25 ± 0.01 ^Ba^	0.20 ± 0.01 ^Ca^
	7	0.80 ± 0.01 ^Aa^	0.78 ± 0.01 ^Aa^	0.81 ± 0.03 ^Aa^	0.80 ± 0.02 ^Aa^	0.24 ± 0.00 ^Bab^	0.20 ± 0.01 ^Ba^
	14	0.81 ± 0.02 ^Aa^	0.79 ± 0.02 ^ABa^	0.76 ± 0.02 ^ABb^	0.73 ± 0.04 ^Bb^	0.23 ± 0.00 ^Cbc^	0.19 ± 0.01 ^Ca^
	21	0.75 ± 0.01 ^Ac^	0.75 ± 0.00 ^Aab^	0.76 ± 0.01 ^Ab^	0.76 ± 0.00 ^Aab^	0.22 ± 0.01 ^Bc^	0.19 ± 0.01 ^Ca^
	28	0.75 ± 0.01 ^Abc^	0.74 ± 0.02 ^Ab^	0.75 ± 0.01 ^Ab^	0.76 ± 0.02 ^Aab^	0.22 ± 0.01 ^Bc^	0.17 ± 0.01 ^Cb^
Total soluble solids (°Brix)	0	5.13 ± 0.12 ^Cc^	7.13 ± 0.25 ^Bc^	7.60 ± 0.17 ^ABb^	7.73 ± 0.06 ^Ab^	7.80 ± 0.26 ^Ac^	7.83 ± 0.21 ^Ab^
	7	6.23 ± 0.40 ^Ba^	8.30 ± 0.17 ^Aa^	8.37 ± 0.12 ^Aa^	8.63 ± 0.42 ^Aa^	8.47 ± 0.06 ^Ab^	8.63 ± 0.12 ^Aa^
	14	6.17 ± 0.21 ^Ca^	8.40 ± 0.44 ^ABa^	8.23 ± 0.32 ^Bab^	8.77 ± 0.06 ^ABa^	8.83 ± 0.06 ^ABa^	9.13 ± 0.31 ^Aa^
	21	6.13 ± 0.06 ^Cab^	8.20 ± 0.36 ^Bab^	8.60 ± 0.36 ^ABa^	8.80 ± 0.26 ^ABa^	8.90 ± 0.00 ^Aa^	9.10 ± 0.17 ^Aa^
	28	5.53 ± 0.23 ^Cbc^	7.47 ± 0.23 ^Bbc^	7.70 ± 0.10 ^ABb^	7.80 ± 0.00 ^ABb^	7.80 ± 0.10 ^ABc^	8.00 ± 0.00 ^Ab^
Total phenolic compounds (mg GAE/L)	0	315.2 ± 24.8 ^ABCa^	259.6 ± 36.4 ^Cc^	322.0 ± 16.8 ^ABCb^	271.7 ± 29.9 ^BCa^	337.9 ± 32.2 ^ABb^	349.2 ± 36.6 ^Aa^
	7	322.7 ± 34.5 ^BCa^	397.0 ± 36.7 ^Aa^	234.3 ± 7.6 ^Dc^	309.8 ± 32.2 ^BCa^	378.8 ± 15.1 ^ABab^	265.9 ± 26.9 ^CDb^
	14	297.0 ± 13.9 ^Ca^	373.7 ± 12.2 ^Aab^	348.5 ± 4.2 ^ABab^	296.2 ± 16.8 ^Ca^	323.2 ± 19.5 ^BCb^	348.5 ± 12.3 ^ABa^
	21	273.7 ± 14.3 ^Ca^	301.0 ± 47.0 ^Cbc^	375.8 ± 26.8 ^ABa^	337.9 ± 17.8 ^BCa^	421.2 ± 26.2 ^Aa^	285.6 ± 18.4 ^Cb^
	28	270.7 ± 12.2 ^BCa^	305.3 ± 22.0 ^ABbc^	237.1 ± 22.0 ^Cc^	320.5 ± 32.2 ^ABa^	336.4 ± 8.0 ^Ab^	303.8 ± 26.7 ^ABab^
Antioxidant activity (µM trolox)	0	77.4 ± 7.3 ^Aa^	66.3 ± 4.8 ^ABCa^	57.3 ± 1.0 ^CDb^	61.9 ± 3.0 ^BCDa^	74.3 ± 4.8 ^ABa^	48.3 ± 4.7 ^Db^
	7	74.9 ± 5.1 ^Aa^	64.0 ± 5.9 ^ABa^	72.9 ± 7.6 ^Aa^	50.6 ± 3.4 ^Cb^	73.3 ± 3.6 ^Aa^	58.2 ± 0.3 ^BCab^
	14	74.0 ± 8.2 ^Aab^	56.3 ± 3.7 ^Ba^	75.0 ± 7.2 ^Aa^	53.4 ± 2.1 ^Bab^	65.4 ± 4.3 ^ABa^	65.2 ± 3.7 ^ABa^
	21	77.6 ± 4.7 ^Aa^	61.4 ± 4.1 ^Ba^	67.9 ± 8.1 ^ABab^	58.6 ± 5.1 ^Bab^	67.2 ± 5.8 ^ABa^	52.2 ± 6.2 ^Bab^
	28	58.6 ± 1.7 ^BCb^	62.8 ± 5.3 ^ABa^	72.8 ± 3.7 ^Aa^	61.4 ± 4.5 ^ABab^	70.3 ± 4.7 ^Aa^	47.7 ± 4.9 ^Cb^

Notes: CON—control juice; PREB—prebiotic; SYN: unfermented symbiotic; SYNf—fermented symbiotic; SYNa—unfermented synbiotic with adjusted pH; SYNfA—fermented synbiotic with adjusted pH. ^A,B,C,D^ Values with the same uppercase letter in a row denotes that the formulations do not differ significantly at the same storage time (*p* < 0.05). ^a,b,c^ Values with the same lowercase letter in a column denotes that the same formulation does not differ significantly over storage time (*p* < 0.05).

**Table 4:** Proximate composition of the yellow mombin beverages.

Parameter	Formulation				
	CON	PREB	SYNf	SYNa	SYNfA
Moisture (g/100 g)	94.7 ± 0.3 ^a^	92.7 ± 0.4 ^b^	92.3 ± 0.6 ^b^	92.8 ± 0.2 ^b^	92.1 ± 0.2 ^b^
Lipid (g/100 g)	ND	ND	1.3 ± 0.6 ^a^	0.6 ± 0.3 ^a^	2.0 ± 0.5 ^a^
Protein (g/100 g)	0.4 ± 0.0 ^a^	0.4 ± 0.2 ^a^	0.4 ± 0.2 ^a^	0.7 ± 0.3 ^a^	0.7 ± 0.1 ^a^
Ash (g/100 g)	0.2 ± 0.0 ^c^	0.2 ± 0.0 ^c^	0.3 ± 0.0 ^b^	0.4 ± 0.0 ^a^	0.4 ± 0.0 ^a^
Fiber (g/100 g) *	0.7	3.0	3.0	3.0	3.0
Carbohydrates (g/100 g) **	4.0	3.7	2.7	2.5	1.8
Caloric value (Kcal/200 mL)	35	33	49	37	56

Notes: CON—control juice; PREB—prebiotic; SYNf—fermented symbiotic; SYNa—unfermented synbiotic with adjusted pH; SYNfA—fermented synbiotic with adjusted pH; ND—not detected. ^a,b,c^ Values with the same lowercase letter in a row denotes that the formulations does not differ significantly (*p* < 0.05). * Fiber value estimated based on the nutritional information of the ingredients. ** Carbohydrate value obtained by difference.

**Table 5:** Color parameters of the yellow mombin beverages.

Parameter	Formulation				
	CON	PREB	SYNf	SYNa	SYNfA
L*	23.95 ± 0.72 ^a^	26.75 ± 2.03 ^a^	24.15 ± 0.67 ^a^	19.29 ± 0.82 ^b^	19.78 ± 0.75 ^b^
a*	−3.84 ± 0.01 ^c^	−4.03 ± 0.22 ^c^	−4.05 ± 0.27 ^c^	−3.06 ± 0.39 ^b^	−2.30 ± 0.15 ^a^
b*	19.20 ± 0.44 ^b^	15.02 ± 1.93 ^c^	17.57 ± 0.98 ^bc^	25.03 ± 1.46 ^a^	26.90 ± 0.46 ^a^
ΔE	-	5.11 ± 3.46 ^ab^	1.84 ± 0.58 ^b^	7.54 ± 1.81 ^a^	8.91 ± 0.69 ^a^

Notes: CON—control juice; PREB—prebiotic; SYNf—fermented symbiotic; SYNa—unfermented synbiotic with adjusted pH; SYNfA—fermented synbiotic with adjusted pH. ^a,b,c^ Values with the same lowercase letter in a row denotes that the formulations does not differ significantly (*p* < 0.05).

**Table 6:** Overall acceptance of selected yellow mombin beverages.

Parameter	Formulation		
	PREB	SYNf	SYNa
Mean acceptance	7.03 ± 1.73	6.79 ± 1.83	4.74 ± 2.14
Median	8.00 ^a^	7.00 ^a^	5.00 ^b^
Acceptability index (%)	78.14	75.46	59.20

Notes: PREB—prebiotic; SYNf—fermented symbiotic; SYNa—unfermented synbiotic with adjusted pH. ^a,b^ Values with the same lowercase letter in a row denotes that the formulations does not differ significantly (*p* < 0.05).

**Table 7:** *L. plantarum* viability (log CFU/mL) in potentially symbiotic beverages before and after simulated gastrointestinal conditions.

Phase	Formulation		
	Control	SYNf	SYNa
Before simulation	8.3 ± 0.0 ^Aab^	9.2 ± 0.2 ^Aa^	8.5 ± 0.6 ^Aa^
Gastric phase	9.0 ± 0.0 ^Aa^	9.4 ± 0.6 ^Aa^	8.8 ± 0.4 ^Aa^
Intestinal phase	7.7 ± 0.1 ^Ab^	8.8 ± 0.7 ^Aa^	8.5 ± 0.6 ^Aa^

Notes: Control—10% inoculum in NaCl 0.9%; SYNf—fermented symbiotic; SYNa—unfermented synbiotic with adjusted pH. ^A^ Values with the same uppercase letter in a row denotes that the formulations do not differ significantly at the digestion phase (*p* < 0.05). ^a,b^ Values with the same lowercase letter in a column denotes that the same formulation does not differ significantly at different digestion phases (*p* < 0.05).

## Data Availability

Data is contained within the article or Supplementary Material.

## Supplementary Materials

The following supporting information can be downloaded at: https://www.mdpi.com/article/10.3390/foods12101994/s1, Table S1: Results of the penalty analysis of formulation PREB; Table S2: Results of the penalty analysis of formulation SYNf.; Table S3: Results of the penalty analysis of formulation SYNa.
