# Supplementary material for: Potentially Synbiotic Yellow Mombin Beverages: Stability during Refrigerated Storage, Physicochemical Characteristics, and Sensory Properties
Source: Foods. 2023 May 15;12(10):1994. doi: 10.3390/foods12101994 (PMC10217551; doi:10.3390/foods12101994)
Supplement: Supplementary file 1 [file foods-12-01994-s001.zip › Table S1 - Supplementary material.pdf]

Supporting Information

for

Potentially synbiotic yellow mombin beverages: Stability during refrigerated storage, physicochemical characteristics and sensory properties

Luís Eduardo Guieu Galvao Telles Ribeiro[a] Leonam da Silva Pereira Batista[b], Cristiane Fernandes de Assis[a,c], Karla Suzanne Florentino da Silva Chaves Damasceno[a,b], Francisco Caninde de Sousa Júnior[a,c]*

[a] *Programa de Pós-graduação em Nutrição, Universidade Federal do Rio Grande do Norte, Av. Senador Salgado Filho, 3000, 59078-970, Natal, RN, Brazil.*

[b] *Departamento de Nutrição, Universidade Federal do Rio Grande do Norte, Av. Senador Salgado Filho, 3000, 59078-970, Natal, RN, Brazil.*

[c] *Departamento de Farmácia, Universidade Federal do Rio Grande do Norte, R. Gal Gustavo Cordeiro de Faria, s/n, Petrópolis, 59012-570, Natal, RN, Brazil.*

***Corresponding author.**

E-mail address: francisco.sousa@ufrn.br (F.C. Sousa Júnior)

Table S1. Results of the penalty analysis of formulation PREB.

Attribute	Level	Percent (%)	Mean drops	p-value	Penalties	p-value
**Acidity**	Too weak	5.49	1.061		1.786	< 0.0001
	JAR	64.84				
	Too strong	29.67	1.920	< 0.0001		
**Sweetness**	Too weak	36.26	1.896	< 0.0001	1.826	< 0.0001
	JAR	52.75				
	Too strong	10.99	1.596			
**Viscosity**	Too weak	8.79	0.567		1.115	0.030
	JAR	85.71				
	Too strong	5.49	1.992			
**Color**	Too weak	1.10	1.102		2.102	0.038
	JAR	96.70				
	Too strong	2.20	2.602			

Significance level of $p < 0.05$.
