# Supplementary material for: Potentially Synbiotic Yellow Mombin Beverages: Stability during Refrigerated Storage, Physicochemical Characteristics, and Sensory Properties
Source: Foods. 2023 May 15;12(10):1994. doi: 10.3390/foods12101994 (PMC10217551; doi:10.3390/foods12101994)
Supplement: Supplementary file 1 [file foods-12-01994-s001.zip › Table S2 - Supplementary material.pdf]

Supporting Information

for

Potentially synbiotic yellow mombin beverages: Stability during refrigerated storage, physicochemical characteristics and sensory properties

Luís Eduardo Guieu Galvao Telles Ribeiro[a] Leonam da Silva Pereira Batista[b], Cristiane Fernandes de Assis[a,c], Karla Suzanne Florentino da Silva Chaves Damasceno[a,b], Francisco Caninde de Sousa Júnior[a,c]*

[a] *Programa de Pós-graduação em Nutrição, Universidade Federal do Rio Grande do Norte, Av. Senador Salgado Filho, 3000, 59078-970, Natal, RN, Brazil.*

[b] *Departamento de Nutrição, Universidade Federal do Rio Grande do Norte, Av. Senador Salgado Filho, 3000, 59078-970, Natal, RN, Brazil.*

[c] *Departamento de Farmácia, Universidade Federal do Rio Grande do Norte, R. Gal Gustavo Cordeiro de Faria, s/n, Petrópolis, 59012-570, Natal, RN, Brazil.*

***Corresponding author.**

E-mail address: francisco.sousa@ufrn.br (F.C. Sousa Júnior)

Table S2. Results of the penalty analysis of formulation SYNf.

Attributes	Level	Percent (%)	Mean drops	*p*-value	Penalties	*p*-value
**Acidity**	Too weak	10.99	2.892		2.398	< 0.0001
	JAR	58.24				
	Too strong	30.77	2.221	< 0.0001		
**Sweetness**	Too weak	39.56	1.321	0.000	1.484	< 0.0001
	JAR	54.95				
	Too strong	5.49	2.660			
**Viscosity**	Too weak	16.48	1.590		1.862	< 0.0001
	JAR	71.43				
	Too strong	12.09	2.232			
**Color**	Too weak	3.30	1.297		1.964	0.003
	JAR	91.21				
	Too strong	5.49	2.364			

Significance level of $p < 0.05$.
