# Supplementary material for: Potentially Synbiotic Yellow Mombin Beverages: Stability during Refrigerated Storage, Physicochemical Characteristics, and Sensory Properties
Source: Foods. 2023 May 15;12(10):1994. doi: 10.3390/foods12101994 (PMC10217551; doi:10.3390/foods12101994)
Supplement: Supplementary file 1 [file foods-12-01994-s001.zip › Table S3 - Supplementary material.pdf]

Supporting Information

for

Potentially synbiotic yellow mombin beverages: Stability during refrigerated storage, physicochemical characteristics and sensory properties

Luís Eduardo Guieu Galvao Telles Ribeiro[a] Leonam da Silva Pereira Batista[b], Cristiane Fernandes de Assis[a,c], Karla Suzanne Florentino da Silva Chaves Damasceno[a,b], Francisco Caninde de Sousa Júnior[a,c]*

[a] *Programa de Pós-graduação em Nutrição, Universidade Federal do Rio Grande do Norte, Av. Senador Salgado Filho, 3000, 59078-970, Natal, RN, Brazil.*

[b] *Departamento de Nutrição, Universidade Federal do Rio Grande do Norte, Av. Senador Salgado Filho, 3000, 59078-970, Natal, RN, Brazil.*

[c] *Departamento de Farmácia, Universidade Federal do Rio Grande do Norte, R. Gal Gustavo Cordeiro de Faria, s/n, Petrópolis, 59012-570, Natal, RN, Brazil.*

***Corresponding author.**

E-mail address: francisco.sousa@ufrn.br (F.C. Sousa Júnior)

Table S3. Results of the penalty analysis of formulation SYNa.

Attribute	Level	Percent (%)	Mean drops	*p*-value	Penalties	*p*-value
Acidity	Too weak	75.82	2.324	< 0.0001	2.268	< 0.0001
	JAR	19.78				
	Too strong	4.40	1.306			
Sweetness	Too weak	27.47	1.602	0.008	2.389	< 0.0001
	JAR	25.27				
	Too strong	47.25	2.847	< 0.0001		
Viscosity	Too weak	14.29	0.554		1.184	0.008
	JAR	43.96				
	Too strong	41.76	1.400	0.005		
Color	Too weak	2.20	1.431		1.581	0.000
	JAR	56.04				
	Too strong	41.76	1.589	0.000		

Significance level of $p < 0.05$.
